# Supplementary material for: Biogeographic Patterns of Fungal Sub-Communities under Different Land-Use Types in Subtropical China
Source: J Fungi (Basel). 2023 Jun 6;9(6):646. doi: 10.3390/jof9060646 (PMC10303538; doi:10.3390/jof9060646)
Supplement: Supplementary file 1 [file jof-09-00646-s001.zip › jof-2398027-supplementary.pdf]

Table S1 Sample Collection Information

Samples	Longitude(E)	Latitude(N)	Vegetation cover	Cultivation History	Category
S1	118.628934	30.820180	paddy field	>10 years	tilled soil
S2	118.649949	30.803800	paddy field	>10	tilled soil
S3	119.272085	30.860087	paddy field	>10	tilled soil
S4	119.517095	30.881888	paddy field	>10	tilled soil
S5	119.188910	31.127631	paddy field	>10	tilled soil
S6	119.038861	30.972781	paddy field	>10	tilled soil
S7	118.158800	30.490595	paddy field	>10	tilled soil
S8	118.432567	30.313183	paddy field	>10	tilled soil
S9	118.603712	30.851279	vegetable field	5	tilled soil
S10	118.623375	30.811664	vegetable field	2	tilled soil
S11	118.671824	30.876421	vegetable field	3	tilled soil
S12	118.813986	31.026375	vegetable field	4	tilled soil
S13	118.929651	31.152928	vegetable field	17	tilled soil
S14	118.678963	30.089233	orchards	7	tilled soil
S15	118.998283	30.690122	orchards	7	tilled soil
S16	118.903259	30.714163	orchards	2	tilled soil
S17	118.599617	30.033812	garden field	10	tilled soil
S18	118.496767	30.175667	garden field	10	tilled soil
S19	118.719741	30.283900	garden field	5	tilled soil
S20	118.952063	30.512156	woodland	>10	untilled soil
S21	118.325851	30.247267	woodland	>10	untilled soil
S22	118.602819	30.839350	woodland	>10	untilled soil
S23	118.600996	30.813893	woodland	>10	untilled soil
S24	119.156651	31.050992	woodland	>10	untilled soil
S25	118.156902	30.486388	woodland	>10	untilled soil
S26	118.506733	30.451498	woodland	>10	untilled soil
S27	119.137628	31.106381	woodland	>10	untilled soil
S28	118.992295	31.211240	woodland	>10	untilled soil
S29	119.032652	31.222129	woodland	>10	untilled soil
S30	118.543230	30.834547	woodland	>10	untilled soil
S31	119.065350	30.484925	woodland	>10	untilled soil
S32	118.678849	30.295851	woodland	>10	untilled soil
S33	119.212328	30.844829	woodland	>10	untilled soil
S34	118.279967	30.570989	dryland	>10	untilled soil
S35	118.425717	30.696039	dryland	>10	untilled soil
S36	119.293499	31.088935	dryland	>10	untilled soil
S37	118.974233	31.215933	dryland	>10	untilled soil
S38	118.438067	30.117149	grassland	>10	untilled soil
S39	118.336612	30.248613	grassland	>10	untilled soil
S40	119.095727	30.532140	grassland	>10	untilled soil
S41	118.554117	30.256951	grassland	>10	untilled soil
S42	118.631885	30.169578	wasteland	>10	untilled soil
S43	118.641683	30.705467	wasteland	>10	untilled soil
S44	118.560569	30.723098	wasteland	>10	untilled soil

Note: Each sampling site was sampled in two layers, with 44 sampling sites and a total of 88 samples.

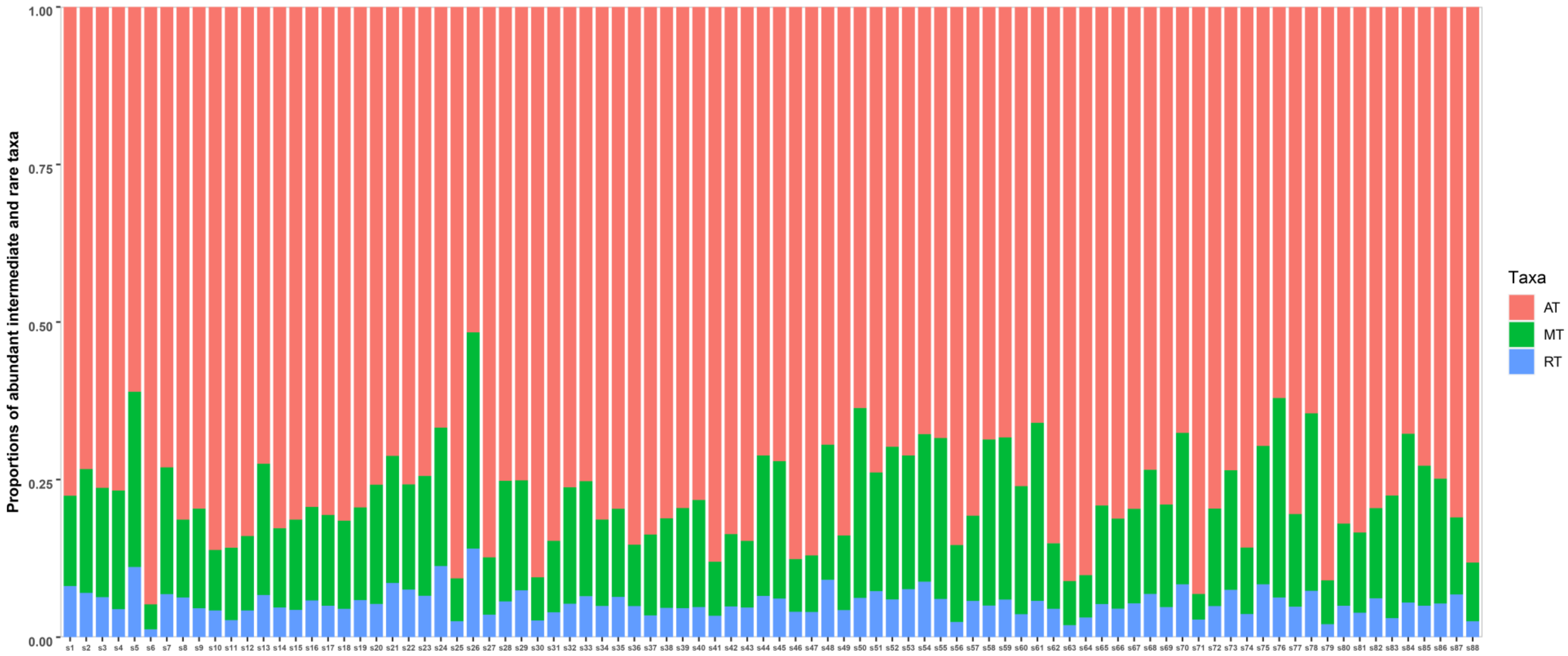

Figure.S1 Proportions of abundant, intermediate and rare taxa in each sample; AT, abundant taxa; MT, intermediate taxa; RT, rare taxa.

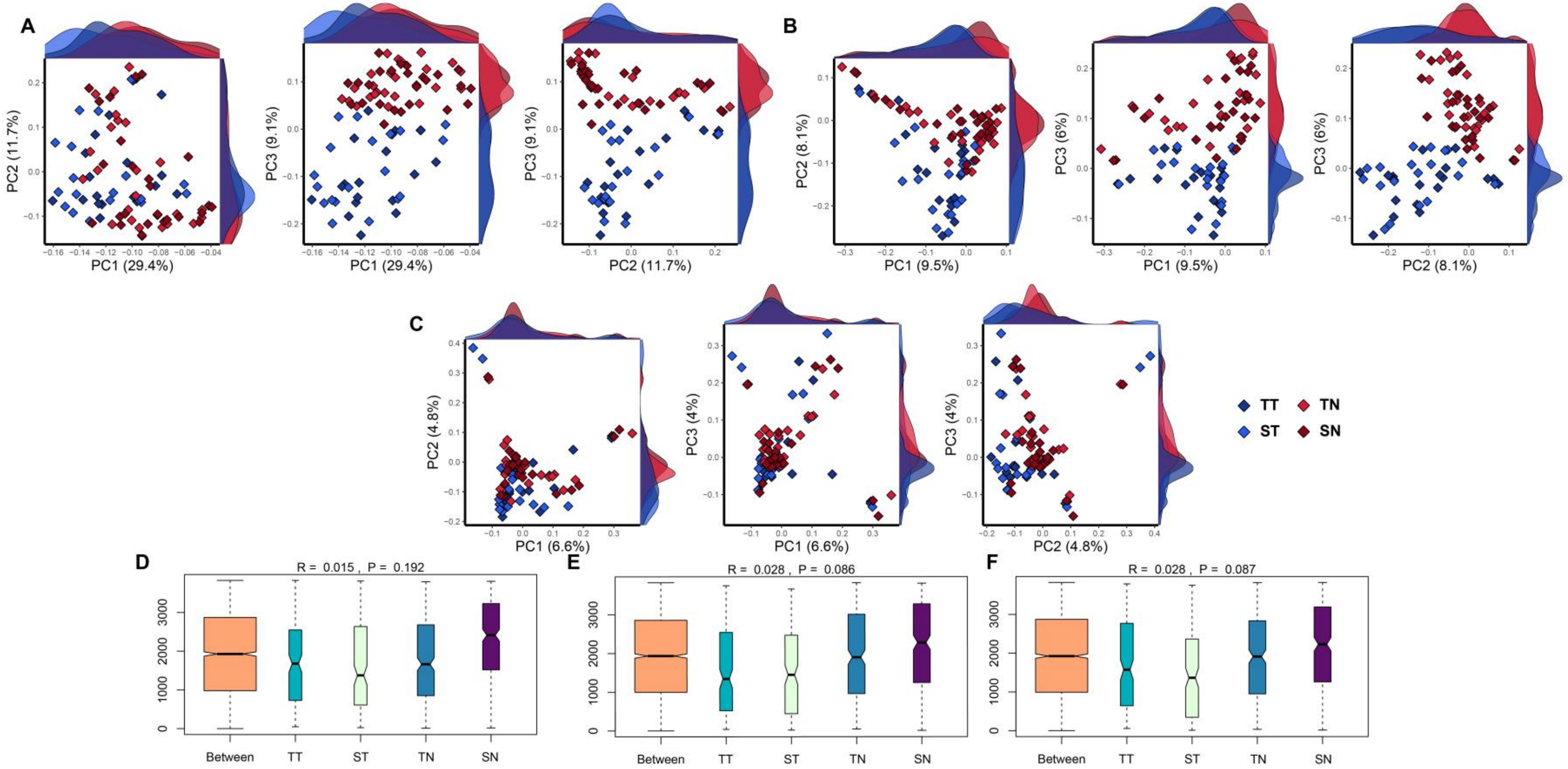

Figure.S2 Differential analysis of the composition of three sub-communities in topsoil and subsoil layers of tilled and untilled lands. PCO analysis for abundant (A), intermediate (B), and rare (C) sub-communities. Analysis of similarities (ANOSIM) for abundant (D), intermediate (E), and rare (F) sub-communities. Tillage topsoil layer, TT; Tillage subsoil layer, ST; No-tillage topsoil layer, TN; No-tillage subsoil layer, SN.

Table S2 Mantel test for fungal similarity and geographical distance

**Treatments**	**R**	***p***
Tilled soils	-0.101	< 0.001
Untilled soils	-0.313	< 0.001
Tilled abundant taxa	-0.096	< 0.001
Untilled abundant taxa	-0.285	< 0.001
Tilled intermediate taxa	-0.101	< 0.001
Untilled intermediate taxa	-0.437	< 0.001
Tilled rare taxa	-0.107	< 0.001
Untilled rare taxa	-0.442	< 0.001

Table S3 Soil properties in different samples

Soil properties	TT			NT		
	Max	Min	Mean	Max	Min	Mean
pH	7.38	4.68	**5.65****	7.29	4.17	**5.28**
TN (g/kg)	2.88	0.76	**1.41***	2.35	0.23	**1.19**
SOM (g/kg)	52.79	12.02	**26.42**^{ns}	74.97	2.54	**26.40**
AP (mg/kg)	228.24	6.69	**63.30*****	85.25	0.08	**9.01**
AK (mg/kg)	973.37	43.35	**220.81*****	204.00	40.71	**93.32**
TP (g/kg)	2.41	0.30	**0.84*****	1.53	0.07	**0.49**
TK (g/kg)	79.41	10.61	**23.35**^{ns}	39.23	8.11	**20.22**

[a]TN, total nitrogen; SOM, soil organic matter; AP, available phosphorus; AK, available potassium; TP, total phosphorus; TK, total potassium;

[b]Soil properties are shown for tillage and non-tillage samples. The maximum value (Max), minimum value (Min) and mean value (Mean) are shown. An asterisk in the Mean columns indicates significant differences between tillage and no-tillage samples, at the 0.001***, 0.01**, and 0.05* levels respectively.
